# Marine systems biology

**DOI:** 10.3389/fgene.2015.00181

**Published:** 2015-05-13

**Authors:** Thierry Tonon, Damien Eveillard

**Affiliations:** ^1^Sorbonne Université, UPMC Univ Paris 06, Centre National de la Recherche Scientifique, UMR 8227, Integrative Biology of Marine Models, Station Biologique de RoscoffRoscoff, France; ^2^LINA UMR 6241, Centre National de la Recherche Scientifique, EMN, Université de NantesNantes, France

**Keywords:** marine model organisms, networks, dynamical modeling, systems ecology, omics analysis, omics integration, regulation, biotic interactions.

The marine environment accounts for an important part of the earth biodiversity, featuring several types of (even extreme) ecosystems, and is at the origin of life. Marine prokaryotic and eukaryotic organisms are very diverse, and are sometimes, in particular for the latter, the result of intricate evolutionary history. This biodiversity, encounters in many different habitats, shapes various types of abiotic and biotic interactions which influence the biology of the organisms and the functioning of ecosystems. To better understand these natural processes, marine scientists historically considered as valuable to gather and interface data obtained at different levels of analysis (molecular, cellular, tissue/organ, organism/individual, population, community, ecosystem), and to conduct interdisciplinary research making use of principles, knowledge and tools of different fields including biology, ecology, mathematics, physics, and more recently computer sciences. This holistic philosophy recently gains a whole new perspective by the emergence of systems biology. Initially motivated by designing new theoretical frameworks, this field quickly showed its interest for understanding the biological systems as a whole while addressing many applied integrative research problems across model species from bacteria to humans.

Because of their interdisciplinary history, marine sciences today follow this momentum by expanding systems biology approaches to a broader range of marine (eco)systems. Not surprisingly, and as already observed in systems biology, these extensions rely on three complementary points. The first overcomes the problem of analyzing high throughput experiments (genomes, transcriptomes, proteomes, metabolomes), the so-called omics. The second integrates heterogeneous datasets within a unique integrative framework, such as networks or dedicated modelings that describe the marine systems of interest. The third emphasizes putative dynamical behaviors of the marine system when submitted to perturbations, via dedicated computational techniques inspired from mathematical and/or logical modeling.

Interestingly, and compared to other natural systems, marine (eco)systems are, by essence, ideal benchmarks for designing new systems biology approaches since (i) as part of the global ocean, these systems are historically considered as multi-scale, and (ii) their nature allows (mostly) to replicate quantitative experiments. Thus, in addition to providing insights into the biology of marine organisms and the functioning of marine ecosystems, these approaches lay the ground for modeling and predicting evolution of marine processes under changing environmental conditions, and also for inspiring and providing some solutions for a bio-based economy. This current Frontiers Research Topic Issue on Marine Systems Biology provides examples of systems biology approaches that cover different methodological aspects and that are applied to marine (eco)systems of variable scale and complexity.

As a first contribution to this volume, Laurent et al. (2014) construct a simple dynamical reaction-based model that simulates the regulation of protein synthesis, and compare their predictions with experimental data to study the importance of protein synthesis during the fertilization in sea urchin. Their results support the involvement of two molecular events in this physiological process: the destabilization of the complex formed by proteins eIF4E (an eukaryotic initiation factor) and 4E-BP (an inhibitor of the initiation of translation), and an increase in the 4E-BP degradation mechanism. Both actions were then shown to be driven by the protein kinase FRAP/mTOR.

Thommen et al. (2015) combine modeling and molecular experiments for studying the effect of light quality on the circadian clock of the phytoplanktonic unicellular green alga *Ostreococcus tauri*. The circadian clock orchestrates a diverse range of cellular functions in multifarious organisms, with light being a major input for the clock monitoring. However, marine organisms may experience large fluctuations in light spectrum due to their distance variations to sea surface. In this context, Thommen et al. assess whether a two component signaling system sensing green and blue light, through RhodHK (a histidine kinase containing a rhodopsin domain) and LOVHK (a second histidine kinase containing a LOV domain) respectively, synchronizes the TOC1–CCA1 central circadian oscillator on a day/night cycle basis. Most of the numerical predictions are in accordance with experimental data, and pave the way for an interesting conclusion showing that *O. tauri*'s clock can be reset by both blue and green light in a relatively independent way. This suggests the marine clock to be plastic enough for adaptation to a wide range of different environments, from open seas to coastal waters.

Two following papers integrate omics data via the reconstruction and analysis of bacterial genome-scale metabolic networks. The first is related to algal-bacterial interactions, a focus of growing interest in marine science and which aims to integrate several species within a unique biological system. Dittami et al. (2014) report on the genome and metabolic capacities of a member of a yet unknown family of Alphaproteobacteria that is closely related to Rhizobiales and frequently lives in association with brown algae, notably the genetic and genomic model *Ectocarpus* sp. A combination of extensive manual annotation and of automatic metabolic network reconstruction is used to decipher the metabolic capacities of this bacterium and generate hypotheses on its putative biotic interactions with *Ectocarpus* sp. This approach lays the ground for interspecies systems biology approaches to study interactions between brown algae and their associated (non-culturable) bacteria, an important step toward understanding the complex system of algae and their symbionts.

Within a different framework, Taffi et al. (2014) describe a computational framework for analyzing effects of bioremediation at the ecosystem level, based on the combination of food web bioaccumulation models and metabolic models of degrading bacteria. As a case study, they consider the bioaccumulation of Polychlorinated Biphenyls (PCBs) in the Adriatic food web, and implement a metabolic model of *Pseudomonas putida* KT2440 (iJN746) extended with the microbial aerobic pathways of PCBs degradation. By using techniques such as Linear Inverse Modeling, Flux Balance Analysis and Ecological Network Analysis, their work opens novel ways for analysis of ecosystems, for *in silico* evaluation of bioremediation strategies, and also provides insights into the ecological role of microbial communities within food webs. As pinpointed by Basler and Simeonidis (2015), such an approach will stimulate new developments in ecological modeling and offer powerful insights into the multi-level interplay among ecosystems, microbial networks and biodegradation.

As a last communication, Hernández-Prieto et al. (2014) review the impact of a range of omics data (genomics, transcriptomics, proteomics, and metabolomics) for understanding marine cyanobacteria. Web-based tools are then proposed to assist researchers in the exploration and meta-analysis of such heterogeneous data, and their benefit is illustrated with a case study, i.e., the identification of conserved elements in regulatory networks based on the analysis of time-series expression data produced for *Prochlorococcus* MED4 and *Synechocystis* PCC 6803 under iron limitation. The authors also comment on some of the limitations related to data quality, data treatment, and currently available algorithms for automatically generated networks.

All these articles aim for a holistic understanding of the marine biological systems without being driven solely by experimental protocols. In this context, systems biology approaches promote either new concepts (i.e., multiple species genome-scale metabolic networks) or new insights (i.e., biological compounds responsible for circadian clock plasticity). However, beyond the combination of cutting edge modeling and omics data analysis, marine systems biology pursues a long tradition of interdisciplinary approaches that will be certainly enriched in the future by studies of new and diverse marine (model) organisms and/or of multi-scale biotic processes that are of interest for the marine realm.

## Conflict of interest statement

The authors declare that the research was conducted in the absence of any commercial or financial relationships that could be construed as a potential conflict of interest.
